# Autochthonous Human and Canine *Strongyloides stercoralis* Infection in Europe: Report of a Human Case in An Italian Teen and Systematic Review of the Literature

**DOI:** 10.3390/pathogens9060439

**Published:** 2020-06-03

**Authors:** Letizia Ottino, Dora Buonfrate, Paola Paradies, Zeno Bisoffi, Alberto Antonelli, Gian Maria Rossolini, Simona Gabrielli, Alessandro Bartoloni, Lorenzo Zammarchi

**Affiliations:** 1 Department of Experimental and Clinical Medicine, University of Florence, 50134 Florence, Italy; letizia.ottino@outlook.com (L.O.); albertoanton88@gmail.com (A.A.); gianmaria.rossolini@unifi.it (G.M.R.); alessandro.bartoloni@unifi.it (A.B.); 2 IRCCS Sacro Cuore Don Calabria Hospital, Negrar, 37024 Verona, Italy; dora.buonfrate@sacrocuore.it (D.B.); zeno.bisoffi@sacrocuore.it (Z.B.); 3 Department of Emergency and Organs Transplantation, Veterinary Section, Campus of Veterinary Medicine, University of Bari, 70124 Bari, Italy; paola.paradies@uniba.it; 4 Microbiology and Virology Unit, Careggi University Hospital, 50134 Florence, Italy; 5 Department of Public Health and Infectious Diseases, Sapienza University of Rome, 00185 Rome, Italy; simona.gabrielli@uniroma1.it; 6 Infectious and Tropical Diseases Unit, Careggi University and Hospital, 50134 Florence, Italy; 7 Referral Center for Tropical Diseases of Tuscany, Careggi University Hospital, 50134 Florence, Italy

**Keywords:** strongyloidiasis, neglected tropical diseases, one health, transplant

## Abstract

Autochthonous human and canine strongyloidiasis is reported in Europe but is unclear whether the transmission of infection still occurs. We report a previously unpublished human case in an Italian teen and perform a systematic review of literature on autochthonous human and canine strongyloidiasis in Europe to investigate the current dynamic of transmission. Overall, 109 papers published after 1987 were included and one previously unpublished Italian case was added. Eighty case reports were retrieved and 42 of them (52.5%) had severe strongyloidiasis. Most cases were diagnosed in Spain, Italy and France. The median age was 58, the most represented age group was 61–70 years, 11 patients were under 30, and 7 of them were diagnosed after 2000. Epidemiological studies on human strongyloidiasis showed prevalence ranging from 0.56% to 28%. Overall, agriculture work, mine work and walking barefoot were the most commonly reported risk factors for infection. Canine strongyloidiasis was reported mainly in Italy (68 cases), but a few cases occurred also in Iceland, Finland, England, Germany, France, Switzerland, Russia, Slovakia, Romania and Greece. Autochthonous strongyloidiasis is still reported in Europe and sporadic transmission still occurs. Health care professionals should be aware of this issue to identify infected subjects and avoid adverse outcomes, especially in immunosuppressed patients. Further investigations are needed to clarify the zoonotic transmission of this nematode.

## 1. Introduction

*Strongyloides stercoralis* is a parasitic nematode able to infect humans, non-human primates, dogs and wild canids. Parasite transmission occurs through direct skin (or mucosae in the case of animals) contact with contaminated soil containing filariform larvae [1]. Transmission through organ transplantation has been also reported in humans and transmammary infection can occur in dogs [2]. The nematode has the unique characteristic of replicate and persists for decades in human or animal hosts because of the so-called autoinfection cycle [3]. In most cases the chronic infection is asymptomatic in both human and animal hosts [4]. Nonspecific gastrointestinal, respiratory or dermatological symptoms may be present, with eosinophilia being frequently detected in humans [1]. In immunocompromised humans, but also in dogs, complicated and potentially fatal clinical syndromes called hyperinfection and disseminated infection may develop [5]. Severe strongyloidiasis has been associated with steroid or other immunosuppressive therapy, solid organ and stem cells transplantation, onco-hematologic malignancy, Human T-cell Leukemia Virus 1 (HTLV-1) and possibly Human Immunodeficiency Virus 1 (HIV-1) [6]. Direct examination of stool has a very low sensitivity for the diagnosis of uncomplicated strongyloidiasis, while in severe infections the larvae can be found almost in any organ or biological sample [7]. More advanced diagnostic methods, such as agar plate culture or polymerase-chain reaction, increase the sensitivity up to about 45 and 57% respectively [8]. Serology has the highest sensitivity (95%), although cross-reactions can cause false positive results in patients with other helminthic infections and false negative results can occur especially in immunosuppressed individuals [8].

Human strongyloidiasis is widespread in tropical and subtropical regions, where the prevalence is between 10% and 40%, rising to 60% in some low socioeconomic areas [6]. Human cases have been also reported in subjects living in temperate areas. Several studies demonstrate a seroprevalence of 0–3.8% in circumscribed areas of the United States [9] and autochthonous cases have been described in Europe, mainly in the northern mining regions and in the Mediterranean area, although is not clear whether transmission is still occurring [6] raising questions on the current maintenance and modality of the parasite lifecycle.

Furthermore, the *Strongyloides* genus includes several pathogens of veterinary importance affecting a wide range of animals worldwide, including companion animals and livestock. Among these, *S. stercoralis* infects both humans and dogs and therefore is potentially zoonotic [4].

*S. stercoralis* dog infection is reported in almost every continent and climatic area [10,11,12,13,14,15]. In the last decades, the zoonotic potential of *S. stercoralis* has become an issue of interest. Some authors have described the presence of *S. stercoralis* antibodies in both humans and dogs living in close contact [16,17], and recent phylogenetic studies [18,19,20] support the idea of a possible cross-transmission of *S. stercoralis* isolates found both in humans and dogs, although transmission between the two hosts was not confirmed [4]. By contrast, studies from Takano Y. et al. excluded a causal relationship between human and canine strongyloidiasis on a Japanese island, where the infection is still endemic [21].

The objective of this paper is to review cases, series and epidemiological studies regarding autochthonous strongyloidiasis in Europe and to report a previously unpublished case that occurred in Italy. The aims of the work are to investigate: i) the occurrence of autochthonous human and canine strongyloidiasis in Europe, ii) the risk factors for autochthonous human uncomplicated and complicated infection and iii) the occurrence of zoonotic transmission in Europe. 

## 2. Materials and Methods

We reported a previously unpublished case of autochthonous *S. stercoralis* infection in an Italian teen and we performed a systematic review of literature on locally acquired human and canine strongyloidiasis in Europe. The systematic review was conducted following the Preferred Reporting Items for Systematic Reviews and Meta-Analyses (PRISMA) guidelines [22]. The review protocol and PRISMA checklist are reported in the Supplementary Materials.

## 3. Case Report 

**Figure 1:**
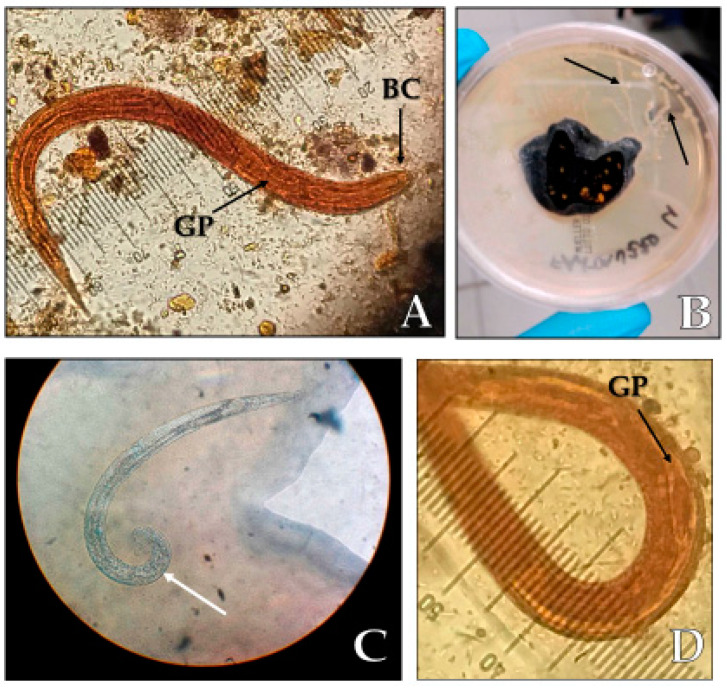
Findings at the agar plate stool culture. (**A)** *Strongyloides stercoralis* rhabditoid larvae L1 (length 250 µm, magnification 40×), BC: buccal cavity, GP: germinal primordium; (**B**) migration paths of the larvae (black arrows) on an agar plate culture; (**C**) *S. stercoralis* adult male with spicule (white arrow); (**D**) germinal primordium.

An 18-year-old Italian girl, born in Tuscany, was referred to our Clinic for Tropical Diseases in 2017 because of a weak positive result of *Trichinella* spp. serology, performed with ELISA technique (NovaTec Immunodiagnostica GmbH, Dietzenbach, Germany), associated with hypereosinophilia lasting for three years. No symptoms were noticeable. The patient never travelled abroad but she used to bathe in Lake Bilancino (Florence province, Tuscany, Italy) and to walk barefoot on the lake shore. Since the age of 14 the complete blood count showed mild anaemia, without eosinophilia, which was treated with oral iron therapy. In 2014, at the age of 15, hypereosinophilia was detected for the first time, with an eosinophil count ranging between 1000 and 1600 cell/mm^3^ in the following years and with no other symptoms associated. In our clinic she underwent further investigations. The examinations showed a normal immunophenotype, negative autoimmunity tests, negative serology for *Trichinella* spp. with Western Blot technique (performed at the Istituto Superiore di Sanità, Rome, Italy). Serology for *Echinococcus* spp. (indirect haemoagglutination Cellognost Echinococcosis IHA, Siemens Healthcare Diagnostics, Marburg, Germany) and *Schistosoma* spp. (Schisto II Western Blot IgG, LD-BIO Diagnostics, Lyon France) were both negative. Surprisingly, a borderline result for *Strongyloides* IgG ELISA (Bordier Affinity Products SA, Crissier, Switzerland) was obtained (index 1, reference values ≤0.9 negative, 0.9–1.1 borderline, ≥1.1 positive) and the diagnosis of strongyloidiasis was confirmed through detection of the rhabdoid larvae with a prominent genital primordium and adult male in agar plate stool culture (Figure 1). Serology for *S. stercoralis* with SeroELISA *Strongyloides* IgG (IVD Research Carlsbad, Canada) was negative (index 0.41, reference value > 1 positive) as well as with the in house Indirect Immune Fluorescent Assay Test previously described by Boscolo et al. (title 0, reference value > 20) [23]. HIV 1–2 Ab/Ag and HTLV 1–2 Ab were negative. PCR for *S. stercoralis* on stool was performed in three different laboratories in Florence (amplification performed with RT-PCR homemade method), in Rome (PCR-based methods targeting the *cox1* gene [24] and Negrar (qPCR performed as described previously by Verweij et al. [25]) and the results were negative. The girl was treated with ivermectin 200 mcg/Kg/day for two days plus another two days of therapy two weeks apart. Blood samples performed after one, six and 12 months showed normal eosinophil count and negative serology for *S. stercoralis* (with the Bordier kit) and agar plate stool culture. 

## 4. Literature Review, Including the Previously Unpublished Case

**Figure 2:**
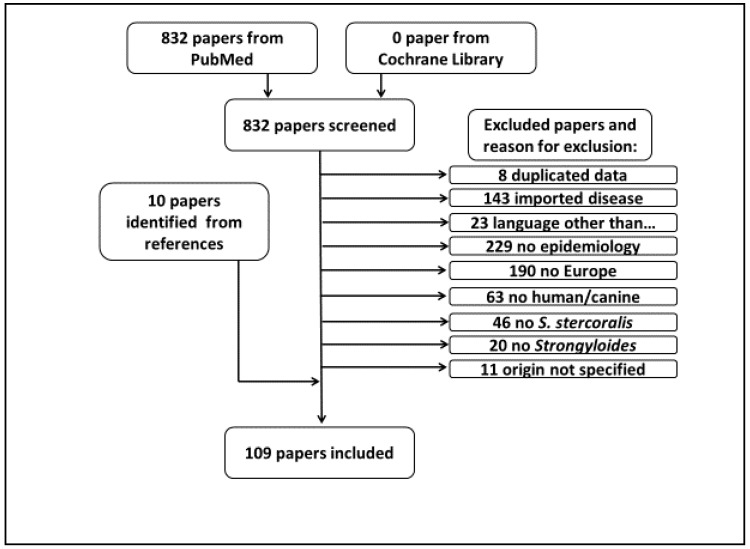
Flow diagram of the selection process.

According to the search strategy, 832 papers were identified, 733 articles were excluded, 10 articles were added from references and 109 paper were eventually included (Figure 2). 

Out of the 109 papers included, 67 were single human strongyloidiasis case reports or case series reporting detailed data on each case, eight were papers reporting human aggregated data, 17 were human epidemiological studies, seven were canine case reports or case series and 11 were canine epidemiological studies. One paper was considered both among canine and human epidemiological studies [16].

**Figure 3:**
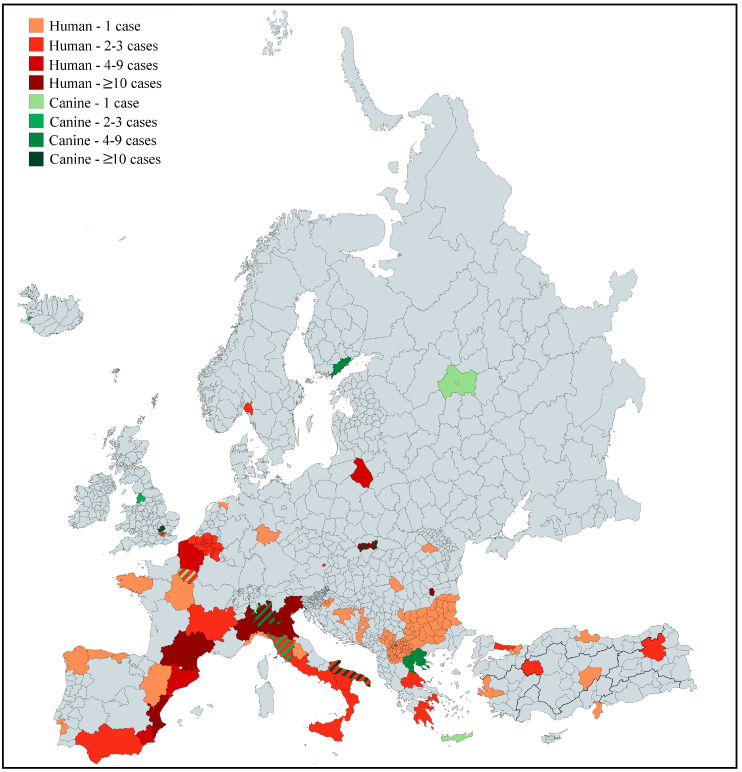
Regional distribution of human (red) and canine (green) autochthonous strongyloidiasis cases in Europe reported in the literature from 1988 to 2018. If multiple countries of exposure were reported, the case was not inserted in the map. One canine case was reported from an unspecified area of Russia and it was arbitrarily highlighted the geographical corresponding to the Russia capital city Moscow [24]. Two countries (Belgium, 2 cases; and Bulgaria, 1 case) were fully coloured since the regions of exposure of respective cases were not specified.

Considering all the kinds of studies included (case reports and case series, paper reporting aggregated data, epidemiological studies), human strongyloidiasis was reported in 19 countries: Austria, Belgium, Bosnia and Herzegovina, Bulgaria, Croatia, England, France, Germany, Greece, Italy, Macedonia, Netherlands, Norway, Poland, Portugal, Romania, Slovakia, Spain and Turkey. The countries where at least 20 human cases were reported were: Spain (565 cases), Italy (264 cases), France (33 cases), Slovakia (25 cases) and Romania (23 cases). Among these countries, the most represented areas were the eastern coast of Spain, especially Valencia province, the north of Italy (Piedmont, Lombardy, Veneto and Friuli-Venezia-Giulia), and the south-west and the north-east of France. Canine strongyloidiasis cases were reported in 12 countries, most of them in Italy (68 cases) and fewer in Belgium, England, Finland, France, Iceland, Germany, Switzerland, Russia, Slovakia, Romania and Greece. The European distribution of human and canine cases, according to the information available from the papers, is illustrated in Figure 3.

### 4.1. Human Case Reports and Case Series

Sixty-seven papers reporting case reports and case series, plus the previously unpublished case reported above, were identified. Overall, 80 autochthonous human strongyloidiasis cases were retrieved in this category. Demographic and clinical details are showed in Table 1 and Table 2. 

**Figure 4:**
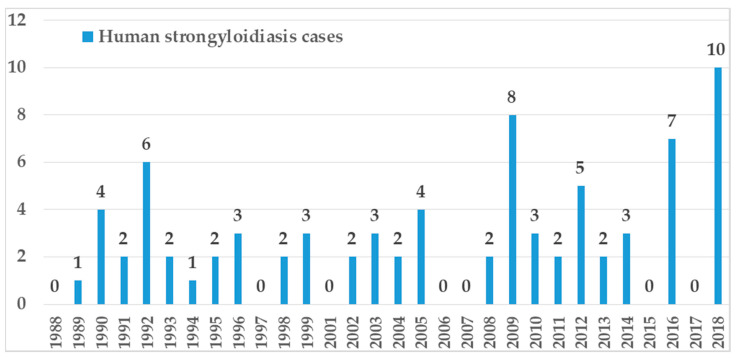
Years of publication of case report/case series on human autochthonous strongyloidiasis in Europe.

Thirty-four papers were published in the decade 2009–2018, 16 papers in the decade 1999–2008, and 17 papers between 1988 and 1998 (Figure 4). 

**Figure 5:**
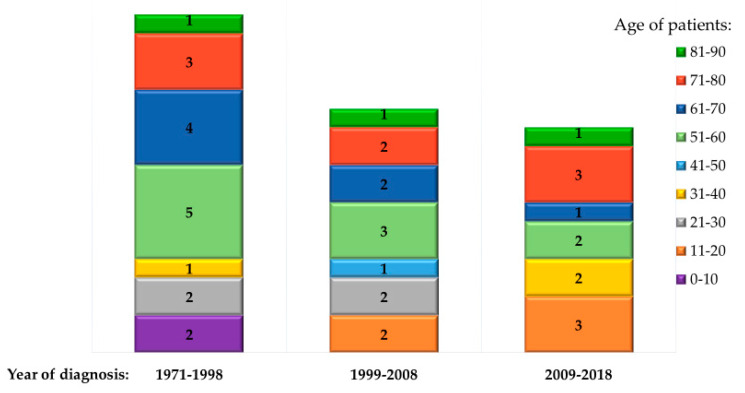
Number and age of patients diagnosed with autochthonous strongyloidiasis in Europe in different periods. Data collected from case reports/case series where the year of diagnosis was available and from an unpublished case reported for the first time in this review.

The year of diagnosis was available in 44 cases: 12 cases were diagnosed in the decade 2009–2018, 13 cases were diagnosed in the decade 1999–2008, and 19 cases were diagnosed from 1971 to 1998 (Figure 5).

Male subjects were 60 (75%) and the median age was 58 years. The most represented age group was between 61 and 70 years (21 patients, 26.2%), 11 patients were under 30 (13.7%), and 7 of them were diagnosed after 2000. Most of the subjects were exposed to infection in Spain (21 cases), followed by Italy (18 cases), France (11 cases) and Turkey (8 cases). Out of the seven patients aged less than 30 years and diagnosed after 2000, two were exposed to infection in Spain, two in Turkey, and one each in France, Italy and Romania. The majority of subjects were symptomatic (72, 90%), as shown in Table 1. Hyperinfection syndrome was recognized in 42 patients (47%), eight of them had a disseminated strongyloidiasis, and 15 had a fatal outcome.

The most frequent risk factor for complicated strongyloidiasis was use of steroid therapy followed by chemotherapy, other immunosuppressive therapy, chronic obstructive pulmonary disease (COPD), oncohematological malignancy or solid tumor, solid organ transplant recipient and HIV infection. Concerning patients with HIV infection and severe strongyloidiasis, six had CD4+ lymphocytes < 60mm^3^ and one had 420 total lymphocytes. Possible factors linked to risk of exposure to strongyloidiasis were reported in 29 subjects: 15 patients were agriculture workers, four were miners, six subjects walked barefoot and two patients had mental disorder; no one was reported to be a dog owner. Interestingly, as it has recently been a matter of debate, two cases of strongyloidiasis in MSM were reported and one of them was supposed to be infected through homosexual intercourse. 

The diagnosis of strongyloidiasis after solid organ transplantation (kidney, pancreas, heart and liver) was done in 12 subjects in Spain (seven cases), Norway (two cases), in Portugal (one case), in Turkey (one case) and in Netherland (one case). Seven transplant recipients most likely acquired the infection through the graft since their donors were originally born in endemic countries and retrospective testing of pre-donation donors serum resulted positive for *S. stercoralis*. In the remaining cases it was not possible to ascertain whether the infection was acquired before the transplant or through the graft.

The most frequently used diagnostic technique was fresh stool examination. Out of 62 patients (77.5%) tested with direct faecal smear examination and/or concentration methods, 47 (75.8%) had a positive result. Details on diagnostic test performed, when available, are listed in Table 2.

### 4.2. Human Aggregated Cases

Data on aggregated human strongyloidiasis cases were obtained from eight papers (Table 3), published between 1992 and 2018. Seven Spanish papers described 61 cases, most of which occurred in the Valencian area, where many of the male subjects had been agriculture workers. Rivasi F. reported 15 cases diagnosed with gastro-duodenal biopsy among a group of immunocompromised subjects living in the Po valley (northern Italy), a region considered endemic for strongyloidiasis. Other two Italian cases have been reported in Florence betwen 2012 and 2014 and the area of exposure was Tuscany and Umbria (central Italy), respectively.

### 4.3. Human Prevalence and Epidemiology Studies

Prevalence data were reported from epidemiological studies conducted at the hospital or community level (Table 4). The prevalence demonstrated a large variability, both within different countries and within different areas of the same country. The highest prevalence has been reported by an Italian paper where 37 out of 132 subjects over 60 (28%) living in the north of Italy (Lombardy and Veneto regions) with eosinophilia had also positive serology (IFAT technique). This was the pilot study of a more extended work by Buonfrate et al. which revealed a prevalence of 8% among subjects over 60 with eosinophilia (cases) and 1% among those without eosinophilia (controls) born in Lombardy, Veneto and Friuli-Venezia-Giulia regions. A prevalence of 7.78% is reported among 120 hospitalized children in Dabrowa Bialostocka District (Poland) with symptoms characteristic to parasite infections. Štrkolcová G. et al. described different prevalence among children and dogs living inside and outside a segregated settlement in the town of Medzev. No children tested positive to stool examination, while serologic test (ELISA technique) showed a prevalence of 33.3% (20/60) among children from settlement and 23.8% (5/21) among children from the rest of the population. Low prevalence is reported in Turkey, where Köksal and colleagues found two out of 27,664 subjects (0.17%) with positive stool samples. This large variability can be explained by both the different diagnostic techniques used, and the different risk factors of the subpopulation analysed in each study.

### 4.4. Canine Strongyloidiasis Case Reports, Aggregated Cases and Prevalence

Scarce literature is available for canine strongyloidiasis in Europe, although the number of reports has increased in the last ten years (Table 5 and Table 6). Most canine strongyloidiasis cases have been reported in southern Italy, in the Apulia region. Iatta et al. assessed the occurrence of *S. stercoralis* infection in dogs living in a kennel in Apulia: out of 100 dogs, 56 scored positive by different diagnostic techniques (serological, coprological and molecular methods). Sporadic infections have been reported in few other European countries. Interestingly a few cases have been described in northern Europe. A case series by Dillard KJ et al. described three infected dogs in a Finnish kennel. Eydal et al. described 20 cases diagnosed from 1989 to 2016 among imported dogs in quarantine (18 of them imported from European countries), and 11 infected household dogs diagnosed from 2012 to 2015 (two Icelandic bitches and nine imported dogs from other European countries). In a prevalence study by Štrkolcová G. et al., the rate of infection detected with the Koga Agar Plate method among dogs living in a Roma settlement in Slovakia was 13.3% (4/30) and 10% (2/20) among dogs living in a shelter. Seroprevalence (with ELISA) in this latter group was 55% (11/20). Low prevalence (between 0% and 3%) is reported in domestic and kennel dogs in England, Germany, Greece and northern Italy and in wild grey wolves in Slovakian natural Parks.

## 5. Discussion

The European countries where most of autochthonous strongyloidiasis cases have been reported are Spain, Italy and France. In Spain, as confirmed by a review by Barroso et al., most cases have been diagnosed in the Valencia region among elderly patients that worked as farmer or in other jobs typically done barefoot [127]. The review showed that the peak of diagnosis occurred from 2001 to 2010, but even if drastically reduced new diagnoses are still reported. In Italy most cases have been reported in the last 20 years, due to prevalence studies conducted among the elderly population living in agricultural areas in the north of the country, where the transmission was frequent in the past. Regarding France, data from a retrospective study by Magnaval et al. suggest that infestation still exists in the Midi-Pyrenees region (southwestern France) [93], while sporadic cases have been reported in almost all other regions from the north to the south. Interestingly, besides the Mediterranean area, a few cases have been described also in colder countries of Eastern Europe, such as Romania, Bulgaria and Poland. Norway is the country at the highest latitude where strongyloidiasis has been described, although the transmission of the infection seemed to be related to kidney transplantation from a donor that was born in an endemic area. The epidemiological findings vary widely among European countries, where the different climate areas and humidity degrees can be more or less favourable for larvae survival in the soil. 

Most of cases were diagnosed with direct stool examination and about half of them were severe cases. Since direct stool examination has a low sensitivity and severe strongyloidiasis develops in a minority of patients, it is probable that there are fewer reported cases compared to the real number of infected people. On the other hand, high seroprevalence reported by some studies, such as the one carried out among Slovakian children with no positive direct stool examinations, should be interpreted with caution since serological false positive results cannot be ruled out [16]. Concerning the transmission of infection through transplantation, we found twelve cases of strongyloidiasis diagnosed after solid organ transplantation in subjects without history of travel outside Europe. All of them were diagnosed in the last decade. In seven cases, the infection was most likely transmitted through the graft since the donors were born in tropical or subtropical endemic countries and resulted positive to *S. stercoralis* serology. In the remaining five cases the infection could have been acquired by the recipient before transplantation or through the graft. 

Canine strongyloidiasis is not frequently reported in Europe. Italy is the country that has recorded the highest number of cases. Epidemiological studies have been conducted in different regions among kennel, wild and domestic dogs, finding prevalence ranging within 3% and 0.2%, with a single study conducted in an Italian shelter reporting a prevalence of 56% [112]. The higher prevalence in the latter study could be due to the diagnostic test performed, since overall prevalence was detected with fecal examination and/or rt-PCR plus serology (ELISA and IFAT). Sporadic canine infections have been described in England, Germany, Greece, Iceland, Romania, Russia, Slovakia and Switzerland.

Concerning human strongyloidiasis case reports, the maximum peak of diagnosis occurred within 60 and 70 years of age from the 1980s to the present. Given the typical chronicity of the infestation, most of these elderly subjects may have been infected in the post-war period when the scarce hygienic conditions could have facilitated the transmission. Although the improvement of health and working conditions probably led to a reduction in transmission, a stronger clinician’s awareness of the disease can explain the stable trend of cases reported. Moreover, some sporadic infections among young patients have been reported, even in the last decade. Several hypotheses can explain these recent cases including soil transmitted cases sustained by persistent free-living cycle in the environment; canine or, less likely, human reservoir; or direct interhuman transmission (for example through sexual intercourse). 

The role of strongyloidiasis as a zoonotic disease has been largely debated [128] under the historical evidence that *S*. *stercoralis* can adapt from human to dog hosts, suggesting that the reverse could also be possible [4,129]. Recent phylogenetic studies [18,19] indicated that *S. stercoralis* consists of two genetically distinct lineages, one isolated from both dogs and humans, the other found exclusively in dogs. Moreover, the study conducted by Jaleta et al. on humans and dogs living in the same household in rural Cambodia demonstrated that dogs carry two genetically different populations of *S. stercoralis* one of which is shared with humans [130]. Thus, this study is the first to provide convincing evidence of natural zoonotic transmission of *S. stercoralis* between dogs and humans. According to this evidence, some authors suggested concomitant treatment of dogs alongside mass drug administration programs to control strongyloidiasis in humans [19,131,132]; however, this approach could be more useful in highly endemic countries rather than in Europe.

Another issue of concern is direct interhuman transmission. Infestation with *S. stercoralis* through homosexual intercourse has been described since 1981 [133], and it has been recognized by the scientific community that sexual contact, especially oral–anal contact, can facilitate the transmission of intestinal parasites [134].

When the disease is not recognized, the characteristic mechanism of autoinfection can lead to chronic infestation persisting for decades, posing infected subjects to ongoing risk of severe manifestations in cases of immunosuppression. According to our findings, strongyloidiasis must be taken in account in elderly European subjects living in endemic areas, with or without clinical symptoms or eosinophilia and whether they are immunosuppressed or a candidate for immunosuppression [135]. The risk of development of a severe form of strongyloidiasis has been associated with many immunosuppressive conditions such as steroid therapy, other immunosuppressants, solid tumour or oncohematologic malignancy, transplantation, and chemotherapy [136]. The COHEMI guidelines by Requena-Méndez et al. provide indications for the screening and management of *Strongyloides* in subjects living in non-endemic countries. Concerning immunosuppressed patients, screening for strongyloidiasis or pre-emptive therapy where a strong clinical suspicious exists is strongly recommended in every subject at risk, including elderly European patients that have lived in endemic areas [137]. In 2013, the American Society of Transplantation provided guidelines recommending the evaluation for *Strongyloides* infection in transplant candidates and donors with epidemiologic risk factors or unexplained eosinophilia during pretransplant evaluation [138]. In our opinion, deeper efforts are needed to improve European screening programs among transplant centers, with a focus on donors’ origins and risk factors for infection. According to this review, cases might occur, although seldom, in young patients. Hence, strongyloidiasis cannot be excluded in young autochthonous patients especially in cases of unexplained eosinophilia or in cases with risk factors such as working as a farmer or miner, walking barefoot, having at risk sexual intercourse, or mental disorders. 

A limitation of our review is the exclusion of grey literature and papers written in languages other than Italian, English, French, Spanish or German, probably underrepresenting the total number of papers. Another limitation is that several cases have probably been misdiagnosed and are not published in the literature. Moreover, a publication bias can be present, due to the higher interest of journals in publishing papers reporting cases of severe strongyloidiasis rather than an asymptomatic infection.

## 6. Conclusions

Traditionally, strongyloidiasis is considered a disease related to tropical and subtropical areas and is often underdiagnosed and underestimated. In Europe transmission of the disease still occurs in humans and dogs, although rarely. Clinicians need to be aware of the epidemiological features of autochthonous infection in order to promptly recognize it and avoid severe complications in immunosuppressed patients. Further studies must be performed to better understand the possible zoonotic transmission of this infection. 

## Figures and Tables

**Table 1:** Demographic and clinical details of 80 patients with autochthonous human strongyloidiasis acquired in Europe described in case reports or case series.

**Demographic Features**	
Median age (range in years)	58 (5–86)
Male sex: n (%)	60 (75%)
**Country of Infection Exposure–[Reference]**	**n (%)**
Belgium [26,27]	2 (2.5%)
Bosnia and Herzegovina [28]	1 (1.2%)
Bulgaria [29]	1 (1.2%)
Croatia [30]	1 (1.2%)
England [31]	1 (1.2%)
France [32,33,34,35,36,37,38,39]	11 (13.7%)
Germany [40]	1 (1.2%)
Greece [41,42,43,44,45]	5 (6.2%)
Italy [46,47,48,49,50,51,52,53,54,55]	18 (22.5%)
Macedonia [56]	1 (1.2%)
Netherlands [57]	1 (1.2%)
Norway [58]	2 (2.5%)
Portugal [59]	1 (1.2%)
Romania [60,61]	2 (2.5%)
Spain [62,63,64,65,66,67,68,69,70,71,72,73,74,75,76]	21 (26.2%)
Turkey [77,78,79,80,81,82,83,84]	8 (10%)
Multiple EU Countries [85,86,87]	3 (3.7%)
**Symptoms among Symptomatic Subjects (n: 72, 90%)**	**n (%)**
Nausea or vomiting	26 (36.1%)
Diarrhea	32 (44.4%)
Abdominal pain	44 (61.1%)
Weight loss	28 (38.9%)
Itching	13 (18%)
Urticaria	13 (18%)
Cough	21 (29.2%)
Dyspnea	13 (18%)
Angioedema	4 (5.5%)
Fever	29 (40.3%)
**Asymptomatic subjects**	8 (10%)
**Eosinophilia**	47 (58.7%)
**Severe Strongyloidiasis**	42 (52.5%)
Hyperinfection Syndrome	42 (52.5%)
Disseminated strongyloidiasis	8 (10%)
**Risk Factors among Patients with Severe Strongyloidiasis**	**n (%)**
Corticosteroid therapy	20 (47.6%)
Chemotherapy	6 (14.3%)
Other immunosuppressive therapy	8 (19%)
COPD	5 (12%)
Oncohematologic disease	3 (7.1%)
Solid tumor	3 (7.1%)
Solid organ transplant recipient	7 (16.7%)
HIV infection	7 (16.7%)
**Reported Risk Factors for Exposure to Infection**	**n (%)**
Agriculture worker	15 (18.7%)
Miners	4 (5%)
Walk barefoot	6 (7.5%)
Mental disorders	2 (2.5%)
Dog owner	0 (0%)
MSM	2 (2.5%)
None reported	51 (63.7%)

Footnotes: EU: Europe, COPD: chronic obstructive pulmonary disease, HIV: human immunodeficency virus, MSM: men who have sex with men.

**Table 2:** Diagnostic test performed in 80 patients with human autochthonous strongyloidiasis in Europe, described in case reports or case series.

Diagnostic Test Performed	n/N (%)
**Agar plate culture** Positive results	**23/80 (28.7%)** 9/23 (39.2%)
**Parasitological test on stool** Positive results	**62/80 (77.5%)** 47/62 (75.8%)
**Positive parasitological test on other specimens**	**22/80 (27.5%)**
Sputum	7/22 (31.8%)
BAL/BA	10/22 (45.4%)
Ascitic fluid/peritoneal dyalisate	2/22 (9%)
Urine	2/22 (9%)
Gastric/jejunal fluid	2/22 (9%)
Bile	1/22 (4.5%)
Urethral smear	1/22 (4.5%)
**Serology**	**19/80 (23.7%)**
Positive results	13/19 (68.4%)
**Histological examination (biopsy/autoptic finding)**	**36/80 (45%)**
Positive gastric/duodenal histologic findings	20/36 (55.5%)
Positive colonic histologic findings	5/36 (13.8%)
Positive liver histologic findings	1/36 (2.7%)
Positive skin histologic findings	2/36 (5.5%)
Positive lung/transbronchial histologic findings	3/36 (8.3%)
**PCR** Positive PCR on stool Positive PCR on BAL	**5/80 (6.2%)** 3/5 (60%) 1/5 (20%)

Footnotes: BAL: bronchoalveolar lavage, BA: bronchial aspirate, PCR: polymerase chain reaction.

**Table 3:** Human strongyloidiasis aggregated data.

First Author and Year of Publication	Type of Study	Diagnostic Test	Country of Exposure	Area of Exposure	N. of Cases	Year of Diagnoses	Age at Diagnosis	Eosinophilia	Symptoms	Hyperinfection Syndrome/ Disseminated Strongyloidiasis	Potential Risk Factors for Infection Reported	Risk Factors for Hyperinfection or Disseminated Infection
Martinez-Perez A 2018 [88]	Retrospective multicentric study on severe strongyloidiasis in Spain	Detection of larvae in fresh stool samples	Spain	Canary Island	1	2006	40	Yes	Digestive, fever, General malaise	Yes	No	Steroids and other immunosuppresssive therapy for renal transplant
Zammarchi L 2017 [89]	Retrospective case series	Pt 1: Serology (ELISA); Pt 2: coprocolture	Italy	Umbria/ Tuscany	2	2012–2014	Pt 1: 70, Pt 2: 23	Pt 1: no, Pt 2: NA	Pt 1: diarrhea, Pt 2: NA	No	Pt 1: no, Pt 2: residency in a nursing institute	Dementia; congenial mental retardation
Geri G 2015 [90]	Case series and review of literature	Parasitological test on stools, respiratory samples, CSF, biopsies	France	France	4	1970–2013	NA	NA	NA	Yes	NA	CCS (n: 111, 83%), immunosuppressive therapy (n: 33. 24.8%), CHT (n: 24. 18.1%), autoimmune disease (n: 33, 24.8%), haematological malignancy, (n: 27, 20.3%), HIV (n: 13, 10.7%)
Rivasi F 2006 [91]	Study on histopathologic alterations of the gastric and duodenal mucosa in Strogyloidiasis	Gastrointestinal biopsy	Italy	Modena (8), Lodi (2), and Novara (5)	15	2000–2005	89–58	NA	NA	NA	NA	Solid tumors (n: 3), hematological malignancy (n: 6), gastric ulcer (n: 1), CCS therapy (n: 1)
Rodríguez Calabuig D 2001 [92]	Case control study to investigate the relationship between occupational activities and strongyloidiasis	Direct stool examination	Spain	Valencia province	45	1997–1999	Mean age 66	NA	23 cough, 22 pruritus, and 13 dyspepsia;	1	Agriculture workers	Corticosteroids (n: 7)
Magnaval JF 2000 [93]	Retrospective descriptive study	Stool examination with Baermann’s method, serology (IFA)	France	Toulouse	17	1991–1997	Mean age 53.9 years (range 25–89 years)	12 patients eosinophil count ≥ 0.6·10^9^cells/L	asymptomatic (n: 7), chronic weakness (n: 5), abdominal pain (n: 4), urticaria (n: 3), diarrhoea (n: 2), muscle pain (n: 2), pruritus (n: 2) flatulence (n: 1), angioedemas (n: 1)	No	Farmers (n: 6), masons (n: 2), swimming pool builder (n: 1), gardener (n: 3)	Alcohol abuse (n: 1)
Rodríguez Calabuig D 1998 [94]	Case series and survey on workplace and domestic health conditions	Fresh stool examination and/or stool culture	Spain/France	Oliva, Valencia province	15 (Spain) + 11 (Spain/France)	1994–1997	Mean age 65	NA	40% respiratory, 26% digestive and 26% dermatologic symptoms	No	66.6% had some risk factor as work barefoot, drink non-potable water	Multiple (COPD, lung cancer, pulmonary fibrosis, duodenal ulcers, diverticulitis, alcoholism, steroid therapy)
Germanaud J 1992 [95]	Annual parasitological coprology survey among members of the hospital kitchen staff	Fresh stool examination, stool culture	France	Orléans	1	1985–1990	NA	NA	NA	NA	NA	NA

N.: number, ELISA: enzyme-linked immunosorbent assay, CSF: cerebrospinal fluid, NA: not available, CCS: corticosteroids, CHT: chemotherapy, IFA: immunofluorescence assay, COPD: chronic obstructive pulmonary disease.

**Table 4:** Human strongyloidiasis epidemiological/prevalence study.

First Author and Year of Publication	Type of Study	Diagnostic Test	Country of Exposure	City of Diagnosis or Where the Study Was Conducted	Level of Data Collection	N. of Cases/ Sample Size	Prevalence	Study Period	Age at Diagnosis	Eosinophilia in Positive Cases	Symptoms/ Hyperinfection Syndrome/ Disseminated Strongyloidiasis	Potential Risk Factors for Infection Reported in Positive Cases	Risk Factors for Hyperinfection or Disseminated Infection in Positive Cases
Winnicki W 2018 [96]	Prevalence study among renal transplant recipients in a division of nephrology and dialysis	Serology (ELISA) (1 duodenal biopsy and direct smear of pleural effusion)	Austria	Wien	Hospital based	6*/200 *(4 patients with possible exposure outside Europe)	3%	NA	Mean age at transplantation 55.9 ± 12.3 years	No	1 patient with hyperinfection	NA	Triple immunosuppressive therapy (tacrolimus, steroids and mycophenolate)
Erdem Kivrak E 2017 [97]	Prevalence study among renal transplant recipients or patients receiving hemodialysis	Serology (ELISA) + RT-PCR (direct microscopy negative)	Turkey	Izmir	Hospital based	1/108	0.92%	2013	NA	NA	No	Walking barefoot	Diabetes mellitus, immunosuppressive therapy for renal transplant
Štrkolcová G 2017 [16]	Prevalence study among Roma segregated settlement and children from general population	Koga agar plate culture, Baermann technique and serology (ELISA)	Slovakia	Medzev	Community based	Fecal sample: 0/60 settlement, 0/21 outside settlement Serology: 20/60 inside settlement, 5/21 outside settlement	Seroprevalence 33.3% children from settlement, 23.8% children from general population	2013–2015	1–17	39 children from segregated settlement and 4 children outside settlement	NA	Children from the settlement: poor hygienic conditions	NA
Buonfrate D 2016 [98]	Epidemiological multicenter case–control study among italian subjects > 60 years old with and without eosinophilia	Serology (ELISA + IFA)	Italy	Negrar, San Bonifacio, Treviso, Brescia, Mantova, Trieste, Udine	Outpatients blood sampling sectors of seven northern Italian hospital	97/1137 among patients with eosinophilia; 13/1178 among patients without eosinophilia	8% among patients with eosinophilia; 1% among patients without eosinophilia	2013–2014	NA	97 patients with eosinophil count ≥500/µL	Pruritus (n: 23), skin rash (n: 13), respiratory symptoms (n: 16), abdominal pain (n: 9), diarrhoea (n: 1)	Age > 60, farm work (n: 32), walking barefoot in earlier years (n: 37)	NA
Valerio L 2013 [99]	Incidence study in north metropolitan area of Barcelona	Any positive test within direct fecal or sputum smear, serology (ELISA), stool culture	Spain	Barcelona	Sentinel clinicians based study	70* newly diagnosed cases in a reference population of 406,000 over a 10-year period *only 2 patients considered autochthonous	0.2 newly diagnosed cases per 10,000 inhabitants per year	2003–2012	NA	NA	NA	NA	NA
Zukiewicz M 2011 [100]	Study on prevalence of different intestinal parasites in children (0–18) with symptoms of possible parasitosis	Microscopic stool test	Poland	Dąbrowa Białostocka	Hospital based	7/120	5.83%	December 2008–May 2009	0–11	NA	NA	NA	NA
Grande R 2011 [101]	Study on prevalence of different intestinal parasites in patients with symptoms of possible parasitosis	Microscopic stool test, agar coproculture and/or serology (ELISA)	Italy	Milan	Hospital based	1/303 children; 4/189 adults	0.33% children; 2.11% adults	2007–2009	NA	NA	NA	NA	NA
Köksal F 2010 [102]	Retrospective prevalence study of intestinal parasites	Faecal concentration technique	Turkey	Istanbul	Hospital based	2/ stool samples of patients with suspicious intestinal parasitic infections	0.17%	1999–2009	NA	NA	NA	NA	NA
Abrescia FF 2009 [103]	Preliminary Epidemiological multicenter case–control study among Italian subjects > 60 years old with and without eosinophilia	Serology (IFAT)	Italy	Mantova- Legnago	Outpatients blood sampling sectors of two northern Italian hospitals	37/132 eligible patients	28%	2008	Mean age 76,4 years, range 68–90 years	100% (eosinophil count >500 cells/µL)	NA	Elderly (born in 1940 or earlier), agricultural area	NA
Pirisi M 2006 [104]	Seroprevalence study among elderly patients with and without eosinophilia	Serology (IFA and ELISA)	Italy	Novara	Hospital based	19/100 patients with eosinophilia and 9/100 patients without eosinophilia	19% patients with eosinophilia and 9% patients without eosinophilia	2002–2003	Mean age 74 years	19 patients with eosinophil count ≥ 0.5·10^9^cells/L	NA	Elderly, agricultural area	NA
Oltra-Alcaraz 2004 [105]	Retrospective, descriptive epidemiological study	Fresh stool examination, Baermann test and fecal culture	Spain	Francesc de Borja Hospital, Area 11 of La Safor, Valencia province	Hospital based, inpatients and outpatients	473/ (261 autochthonous)	0.33%	1995–1999	21–100 years old	NA	NA	Agriculture activities (n: 124), irrigation ditches cleaners (n: 33), ditch baths (n: 6), construction activities (n: 18)	NA
Román-Sánchez P 2003 [106]	Prevalence study among farm workers and analysis of predictive factors of infection	Agar plate culture technique	Spain	Gandìa	Community based	31/250 farm workers	12.4%	2003	Mean age 68.6 years	NA	NA	Farm workers, (100%), history of working barefoot (97%)	Alcohol (19.3%), Smoking (74.2%), Gastrectomy (3.2%), Debilitating illness (22.5%), Corticoids (3.1%), Immunosuppressors (3.1%)
Román-Sánchez P 2001 [107]	Epidemiological prospective study	Direct fecal examination	Spain	Gandìa	Hospital based	152/patients admitted to the hospital	0.9%	1990–1997	Mean age 67 years	82% (n: 125) eosinophil count > 500 cells/mm^3^	Asymptomatic 41.65% (n: 63), Severe infection 13% (n: 20)	Agriculture workers	COPD (n: 44), heart disease (n: 38), solid neoplasia (n: 7), HIV infection (n: 1)
Gatti S 2000 [108]	Prevalence study of parasitic infections in an institution for mentally retarded	Microscopic fecal examination	Italy	Mogliano Veneto	Hospital based	1/550 patients	0.8%	NA	NA	NA	NA	Mental retardation	NA
Cremades Romero MJ, Igual Adell R 1997 [109]	Observational and descriptive prospective study	Fresh stool examination and/or fecal culture, bronchoalveolar lavage and gastric biopsy	Spain	Valencia province	Hospital Comarcal Francesc de Borja de Gandìa, inpatients and outpatients	37	NA	1994–1995	Mean age 68 years (range 51–87 years)	65% had respiratory, digestive and/or cutaneous symptoms	Asymptomatic (n: 13), pruritus (n: 14), flatulence (n: 8), pyrosis (n: 6), epigastralgia (n: 5), nausea and vomit (n: 3), diarrhoea (n: 2), constipation (n: 2), gastrointestinal bleeding (n: 1), skin lesions (n: 2), respiratory symptoms (n: 4), meningitis (n: 1)	Farmers (n: 33)	Solid tumor (n: 2), gastrectomy (n: 1), diabetes (n: 3), alcoholic (n: 1), COPD (n: 9), asthma (n: 9)
Panaitescu D 1995 [110]	Prevalence study of parasitosis in children with handicaps	Agar plate culture	Romania	Bucharest	Hospital based	21/231	9.9%	NA	3–6 years	NA	NA	Children with physical and psychic handicaps	NA
Genta RM 1988 [111]	Prevalence study among inpatients and outpatients of Infectious Disease Department in San Matteo Hospital	Formol-ether stool concentration, coprocolture	Italy	Pavia	Hospital based	118/4203 (48 certainly autochthonous)	3%	1984–1986	Mean age 61.4 years (range 38–80 years)	43 patients (90%) with eosinophils >5%	Gastrointestinal manifestation (n: 23; 47%), skin rash (n: 22; 46%)	Living in endemic area	Steroid treatment (n: 1), previous gastric surgery (n: 7)

NA: not available, ELISA: enzyme-linked immunosorbent assay, RT-PCR: reverse transcription polymerase chain reaction, IFA: immunofluorescence assay, IFAT: immunofluorescence antibody test, COPD: chronic obstructive pulmonary disease.

**Table 5:** Canine strongyloidiasis prevalence/epidemiological data.

First Author and Year of Publication	Type of Study	Diagnostic Test	Country of Exposure	City of Diagnosis	Setting of Data Collection	N. of Cases/Sample Size	Prevalence	Study Period
Iatta R 2018 [112]	Prevalence study and comparative study between different diagnostic tests	Coprological methods, rt-PCR on stool, serology (ELISA/IFAT)	Italy	Bari	Shelter	36/100 (coprocolture and/or rt-PCR); 56/100 (serology)	36% (coprological test and/or rt-PCR on feces) and 56% (adding serology)	2017
Čabanová V 2017 [113]	Epidemiologic parasitological study	Fresh stool examination (flotation method with zinc sulphate)	Slovakia	Košice	Tatra National Park, Muránska Planina National Park and Pol’ana Protected Landscape Area	NA (2 stool samples)	NA (0.8% fecal samples)	2015–2016
Sauda F 2017 [114]	Prevalence study on *Leishmania infantum*, *Dirofilaria spp*. and other endoparasite	Fresh stool examination, Baermann method	Italy	NA	Kennels in Latium and Tuscany	Not available	0.2%	2011–2014
Štrkolcová G 2017 [16]	Prevalence study	Microscopic stool sample (KAP culture, Baermann method) and serology (ELISA)	Slovakia	Medzev	Segregated settlement and shelter	Fecal sample: 4/30 dogs in settlement, 2/20 dogs in shelter outside settlement (KAP method); 0 (Baermann method) Serology: Not performed in dogs inside settlement. 11/20 dogs in shelter outside settlement	Fecal sample: 13.3% settlement, 10% shelter outside settlement (KAP method); 0% (Baermann method) Serology: not performed in dogs inside settlement 55% in dogs in shelter outside settlement	2013–2015
Kostopoulou D [115] 2017	Prevalence study of intestinal parasites in cats and dogs in Crete	Sedimentation, flotation, Telemann sedimentation	Greece	Crete	Household/ shelter/ shepherd dogs	1/879	0.1%	2011–2015
Wright I 2016 [116]	Prevalence study of intestinal nematode in cats and dogs	FLOTAC method	England	Lancashire	Domestic dogs	3/171	1.7%	NA
Zanzani SA 2014 [117]	Prevalence study of intestinal parasites	Coproscopy	Italy	Milan	Fecal samples collected in the metropolitan area of Milan	4/463 fecal samples collected	0.86%	2010
Riggio F 2013 [14]	Prevalence study of intestinal and lung parasites in cats and dogs	Coproscopy/Baermann method	Italy	Pisa	Domestic dogs	2/239	0.8%	2008–2010
Mircean V 2012 [118]	Prevalence study of *G. intestinalis* infection	Fresh stool examination	Romania	Cluj-Napoca	Kennel/shelters/shepherd/household dogs from West, North-West, Center and North-East Romania	2/614	NA	2008/2009
Papazahariadou M 2007 [119]	Prevalence study of gastrointestinal parasites of shepherd and hunting dogs	Telemann’s sedimentation method	Greece	Thessaloniki	Owned dogs	1 shepherd and 4 hunting dogs/281	1.8% (0.7% shepherds, 2.4% hunting dogs)	2004–2005
Gothe R 1990 [120]	Incidence study on endoparasites of bitches and their puppies	Fecal flotation	Germany	Southern Germany	Bitches and their puppies in Southern Germany	NA	3%	NA

Rt-PCR: reverse transcriptase polymerase chain reaction, ELISA: enzyme-linked immunosorbent assay, IFAT: immunofluorescence antibody test, NA: not available, KAP: Koga agar plate.

**Table 6:** Canine strongyloidiasis case report/case series.

First Author and Year of Publication	Type of Study	N. of Cases	Diagnostic Test	Country of Exposure	City of Diagnosis	Wild/Kennel /Domestic Dog	Year of Diagnosis	Age of Dog	Symptoms	Severe Infection	Eosinophilia	Fatal Outcome
Basso W 2018 [113]	Case series	3	Sedimentation-flotation, Baermann method+/- SAFC	Belgium/ France/Switzerland	Bern	Domestic dog (n: 2), Kennel dog (n: 1)	NA	< 11 months	Gastro-intestinal signs (n: 3); itching, cough (n: 1)	No (n: 3)	NA	No (n: 3)
Cvetkovikj A 2018 [121]	Case report	1	Flotation, Baermann method	Russia	Skopje	Domestic dog	2017	6 months	Diarrhea, weight loss	No	No	No
Cervone M 2016 [122]	Case report	1	Flotation, Baermann method	France	Paris	Domestic dog	NA	10 months	Diarrhea, weight loss, abdominal pain, vomiting, itching, cough	Yes	No	No
Paradies P 2017 [123]	Case series	6	Coproscopy/Baermann method	Italy	Bari	Shelter dog (n: 5), domestic dog (n: 1)	NA	adult	Acute (n: 1) or Chronic (n: 4) gastro-intestinal signs +/- depression (n: 2), abdominal mass (n: 1). No symptoms (n: 1)	No (n: 6)	No (n: 5), Mild (n: 1)	No (n: 5); Yes (n: 1)
Eydal M 2016 [124]	Screening study	2 autochthonous household dogs + 27 imported dogs from Europe	Fresh stool examination (formalin-ethyl acetate sedimentation technique or Baermann method)	Iceland/ imported dogs	Reykjavík	Kennel and domestic dogs	1989–2016		Asymptomatic and symptomatic (soft stool, diarrhoea, blood in feces	No	NA	No
Dillard KJ 2007 [125]	Case series	4	Intestinal autoptic findings (n: 1); Baermann method (n: 3)	Finland	Helsinki	Kennel dog	NA	10 weeks (n: 1); NA (n: 3)	Severe gastro-intestinal signs (n: 1), no symptoms (n: 3)	Yes (n: 1), No (n: 3)	NA	Yes (n: 1), No (n: 3)
Gibbons LM 1988 [126]	Case series	20	autoptic findings on gastrointestinal mucosa	England	NA	Kennel dogs	NA	NA	No	No	NA	NA

NA: not available, SAFC: sodium acetate-acetic acid-formalin-concentration.

## Supplementary Materials

The following are available online at https://www.mdpi.com/2076-0817/9/6/439/s1. Supplementary Material 1: Review Protocol, Table S1: PRISMA checklist.
