# Supplementary material for: Autochthonous Human and Canine Strongyloides stercoralis Infection in Europe: Report of a Human Case in An Italian Teen and Systematic Review of the Literature
Source: Pathogens. 2020 Jun 3;9(6):439. doi: 10.3390/pathogens9060439 (PMC7350350; doi:10.3390/pathogens9060439)
Supplement: Supplementary file 1 [file pathogens-09-00439-s001.pdf]

pathogens

MDPI

Article title: Autochthonous human and canine *Strongyloides stercoralis* infection in Europe: report of a human case in an Italian teen and systematic review of the literature

Authors: Letizia Ottino et al

Supplementary material 1: Review Protocol

Case reports, case series and epidemiological or prevalence studies reporting original data on laboratory confirmed cases of human or canine strongyloidiasis acquired in Europe were considered eligible for inclusion. The research was conducted in the online database PubMed (http://www.ncbi.nlm.nih.gov/pubmed/) and The Cochrane Library (https://www.cochranelibrary.com/) using a free text search as follows: (Strongyloides OR strongyloidiasis OR anguillulosis OR anguilluliasis OR strongyloidiasis OR stercoralis) AND (Italy OR Spain OR Austria OR Belgium OR Bulgaria OR Cyprus OR Czech Republic OR Denmark OR Estonia OR Finland OR France OR Germany OR Greece OR Hungary OR Ireland OR Latvia OR Lithuania OR Luxemburg OR Malta OR Netherlands OR Poland OR Portugal OR Romania OR Slovakia OR Slovenia OR Spain OR Sweden OR United Kingdom OR Switzerland OR Norway OR Russia OR Croatia OR Montenegro OR Iceland OR Turkey OR Macedonia OR Europe). The search strategy was conducted on the 29th of December 2018. Two authors (LO, LZ) screened titles and abstracts and selected the studies, inter-reviewer disagreements were solved by discussion.

Papers retrieved were screened according to their title or abstract. Thereafter a second screening was performed by reading the whole full text. If essential data were missing in the full text, they were sought with an attempt to contact the authors. The papers were eventually included or excluded whereas an exhaustive answer was obtained.

Inclusion criteria were: i) case reports, case series, epidemiologic or prevalence studies about human or canine strongyloidiasis; ii) infection acquired in one of the following European countries Austria, Belgium, Bulgaria, Croatia, Cyprus, Czech Republic, Denmark, Estonia, Finland, France, Germany, Greece, Hungary, Iceland, Ireland, Italy, Latvia, Lithuania, Luxemburg, Macedonia, Malta, Montenegro, Netherlands, Norway, Poland, Portugal, Romania, Russia, Slovakia, Slovenia, Spain, Sweden, Switzerland, Turkey, United Kingdom; iii) strongyloidiasis diagnosed by: serology performed with any method, detection of larvae in stool samples or in any body fluid, stool cultures, PCR (Polymerase Chain Reaction) on stool or in any body fluid, histological or autopsy findings.

Exclusion criteria were: i) papers written in languages other than English, Italian, German, French and Spanish; ii) articles published before 1988; iii) duplicated data; iv) imported disease from all continents except Europe; v) papers where patient origin was not specified; vi) papers where was not possible to find out if the patient had previously travelled in or emigrated from an extra-European country endemic for strongyloidiasis; vii) infection caused by *Strongyloides* spp. other than "*stercoralis*" or by other parasites/nematodes. The data were extracted using an Excel database.

Human and canine strongyloidiasis papers were separated in two different groups. Human strongyloidiasis papers were divided into “case reports/case series”, where clinical and demographical data were available, “aggregated cases” where individual data were not available, and “epidemiologic or prevalence studies”. Canine strongyloidiasis papers were divided into “case reports/case series” and “epidemiological or prevalence studies”.

pathogens

MDPI

Article title: Autochthonous human and canine *Strongyloides stercoralis* infection in Europe: report of a human case in an Italian teen and systematic review of the literature

Authors: Letizia Ottino et al

Table S1: PRISMA checklist

Section/topic	#	Checklist item	Reported on page #
**TITLE**			
Title	1	Identify the report as a systematic review, meta-analysis, or both.	1
**ABSTRACT**			
Structured summary	2	Provide a structured summary including, as applicable: background; objectives; data sources; study eligibility criteria, participants, and interventions; study appraisal and synthesis methods; results; limitations; conclusions and implications of key findings; systematic review registration number.	1
**INTRODUCTION**			
Rationale	3	Describe the rationale for the review in the context of what is already known.	2
Objectives	4	Provide an explicit statement of questions being addressed with reference to participants, interventions, comparisons, outcomes, and study design (PICOS).	2
**METHODS**			
Protocol and registration	5	Indicate if a review protocol exists, if and where it can be accessed (e.g., Web address), and, if available, provide registration information including registration number.	Annex
Eligibility criteria	6	Specify study characteristics (e.g., PICOS, length of follow-up) and report characteristics (e.g., years considered, language, publication status) used as criteria for eligibility, giving rationale.	Annex
Information sources	7	Describe all information sources (e.g., databases with dates of coverage, contact with study authors to identify additional studies) in the search and date last searched.	Annex

pathogens

MDPI

Article title: Autochthonous human and canine *Strongyloides stercoralis* infection in Europe: report of a human case in an Italian teen and systematic review of the literature

Authors: Letizia Ottino et al

Search	8	Present full electronic search strategy for at least one database, including any limits used, such that it could be repeated.	Annex
Study selection	9	State the process for selecting studies (i.e., screening, eligibility, included in systematic review, and, if applicable, included in the meta-analysis).	Annex
Data collection process	10	Describe method of data extraction from reports (e.g., piloted forms, independently, in duplicate) and any processes for obtaining and confirming data from investigators.	Annex
Data items	11	List and define all variables for which data were sought (e.g., PICOS, funding sources) and any assumptions and simplifications made.	Annex
Risk of bias in individual studies	12	Describe methods used for assessing risk of bias of individual studies (including specification of whether this was done at the study or outcome level), and how this information is to be used in any data synthesis.	Annex
Summary measures	13	State the principal summary measures (e.g., risk ratio, difference in means).	Annex
Synthesis of results	14	Describe the methods of handling data and combining results of studies, if done, including measures of consistency (e.g., I^2) for each meta-analysis.	Annex
Risk of bias across studies	15	Specify any assessment of risk of bias that may affect the cumulative evidence (e.g., publication bias, selective reporting within studies).	Annex
Additional analyses	16	Describe methods of additional analyses (e.g., sensitivity or subgroup analyses, meta-regression), if done, indicating which were pre-specified.	Annex
**RESULTS**			
Study selection	17	Give numbers of studies screened, assessed for eligibility, and included in the review, with reasons for exclusions at each stage, ideally with a flow diagram.	4-5
Study characteristics	18	For each study, present characteristics for which data were extracted (e.g., study size, PICOS, follow-up period) and provide the citations.	6-7, 11, 13-15, 17-18

Article title: Autochthonous human and canine *Strongyloides stercoralis* infection in Europe: report of a human case in an Italian teen and systematic review of the literature

Authors: Letizia Ottino et al

Risk of bias within studies	19	Present data on risk of bias of each study and, if available, any outcome level assessment (see item 12).	20
Results of individual studies	20	For all outcomes considered (benefits or harms), present, for each study: (a) simple summary data for each intervention group (b) effect estimates and confidence intervals, ideally with a forest plot.	6-9, 11, 13-15, 1617
Synthesis of results	21	Present results of each meta-analysis done, including confidence intervals and measures of consistency.	4-18
Risk of bias across studies	22	Present results of any assessment of risk of bias across studies (see Item 15).	20
Additional analysis	23	Give results of additional analyses, if done (e.g., sensitivity or subgroup analyses, meta-regression [see Item 16]).	-
**DISCUSSION**			
Summary of evidence	24	Summarize the main findings including the strength of evidence for each main outcome; consider their relevance to key groups (e.g., healthcare providers, users, and policy makers).	19-20
Limitations	25	Discuss limitations at study and outcome level (e.g., risk of bias), and at review-level (e.g., incomplete retrieval of identified research, reporting bias).	20
Conclusions	26	Provide a general interpretation of the results in the context of other evidence, and implications for future research.	20
**FUNDING**			
Funding	27	Describe sources of funding for the systematic review and other support (e.g., supply of data); role of funders for the systematic review.	20

pathogens

MDPI

Article title: Autochthonous human and canine *Strongyloides stercoralis* infection in Europe: report of a human case in an Italian teen and systematic review of the literature

Authors: Letizia Ottino et al
